# [Corrigendum] lncRNA SNHG3 acts as oncogene in ovarian cancer through miR‑139‑5p and Notch1

**DOI:** 10.3892/ol.2025.15026

**Published:** 2025-04-08

**Authors:** Li Zhang, Guihua Li, Xiuzhen Wang, Youli Zhang, Xia Huang, Huazhen Wu

Oncol Lett 21: 122, 2021; DOI: 10.3892/ol.2020.12383

Following the publication of the above article, an interested reader drew to the authors’ attention that some of the data panels shown in the scratch wound assays in Fig. 4C and 6C appeared to be overlapping, such that the data may have been derived from a smaller group of original data resources. The authors have re-examined their original data, and realized that a number of errors were made during the compilation of these figures.

The corrected versions of Figs. 4 and 6 are shown on the next page, featuring data from repeated scratch wound assay experiments for Figs. 4C and 6C. Note that the revisions made to these figures do not affect the overall conclusions reported in the paper. All the authors agree to the publication of this corrigendum. The authors are grateful to the Editor of *Oncology Letters* for allowing them the opportunity to publish this Corrigendum, and apologize to the readership for any inconvenience caused.

## Figures and Tables

**Figure 4.**
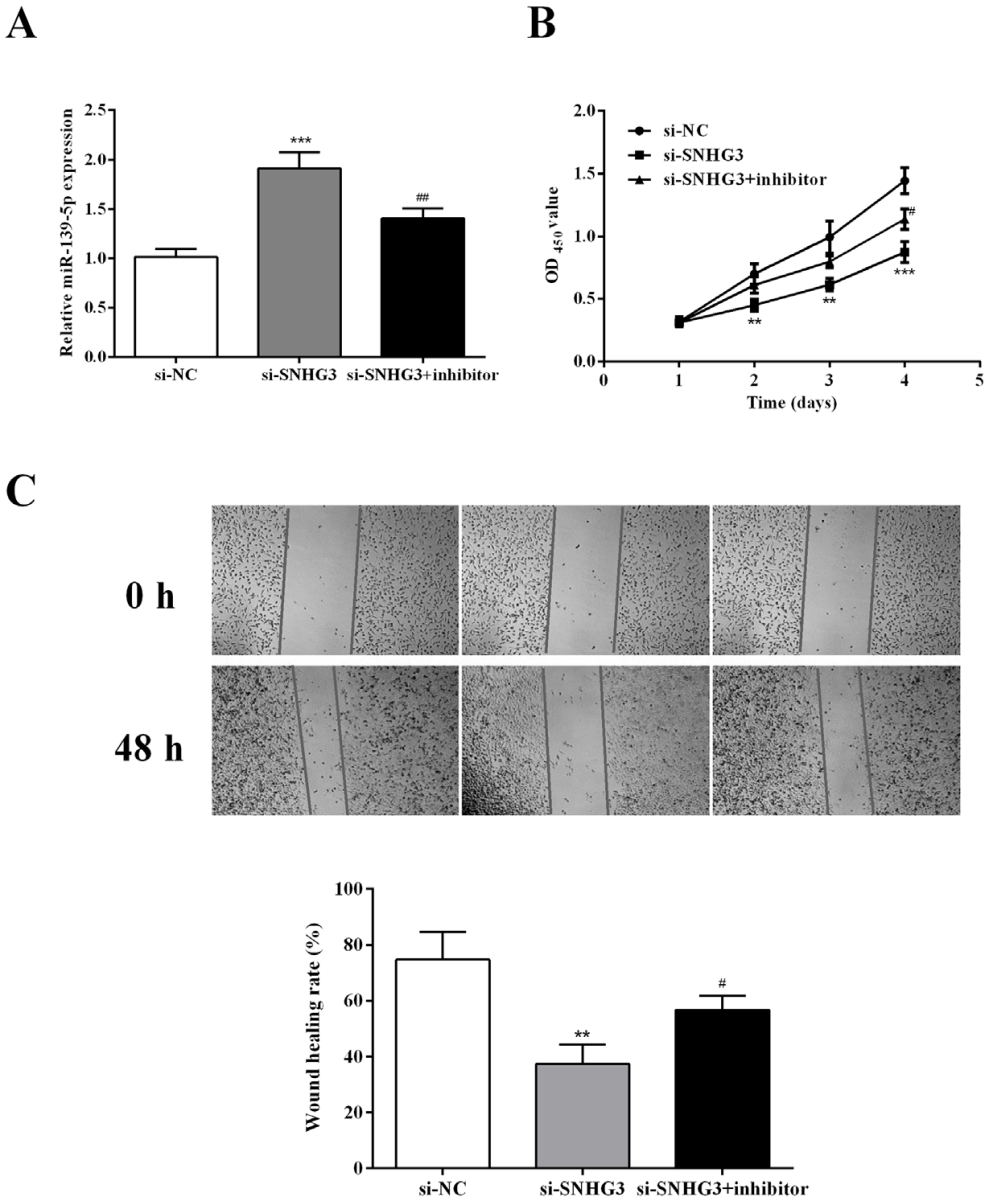
Inhibition of miR-139-5p reverses the suppressive effect of SNHG3 silencing on proliferation and migration. (A) miR-139-5p expression was detected by RT-qPCR in OVCAR3 cells after SNHG3 and miR-139-5p inhibition. (B and C) CCK-8 and wound-healing assays were conducted to detect cell proliferation and migration in OVCAR3 cells with SNHG3 and miR-139-5p inhibition. **P<0.01 and ***P<0.001 compared with si-NC; ^#^P<0.05 and ^##^P<0.001 compared with the si-SNHG3 group. miR, microRNA; SNHG3, small nucleolar RNA host gene 3; si-SNHG3, small interfering RNA targeting SNHG3; CCK-8, Cell Counting Kit-8; NC, negative control.

**Figure 6.**
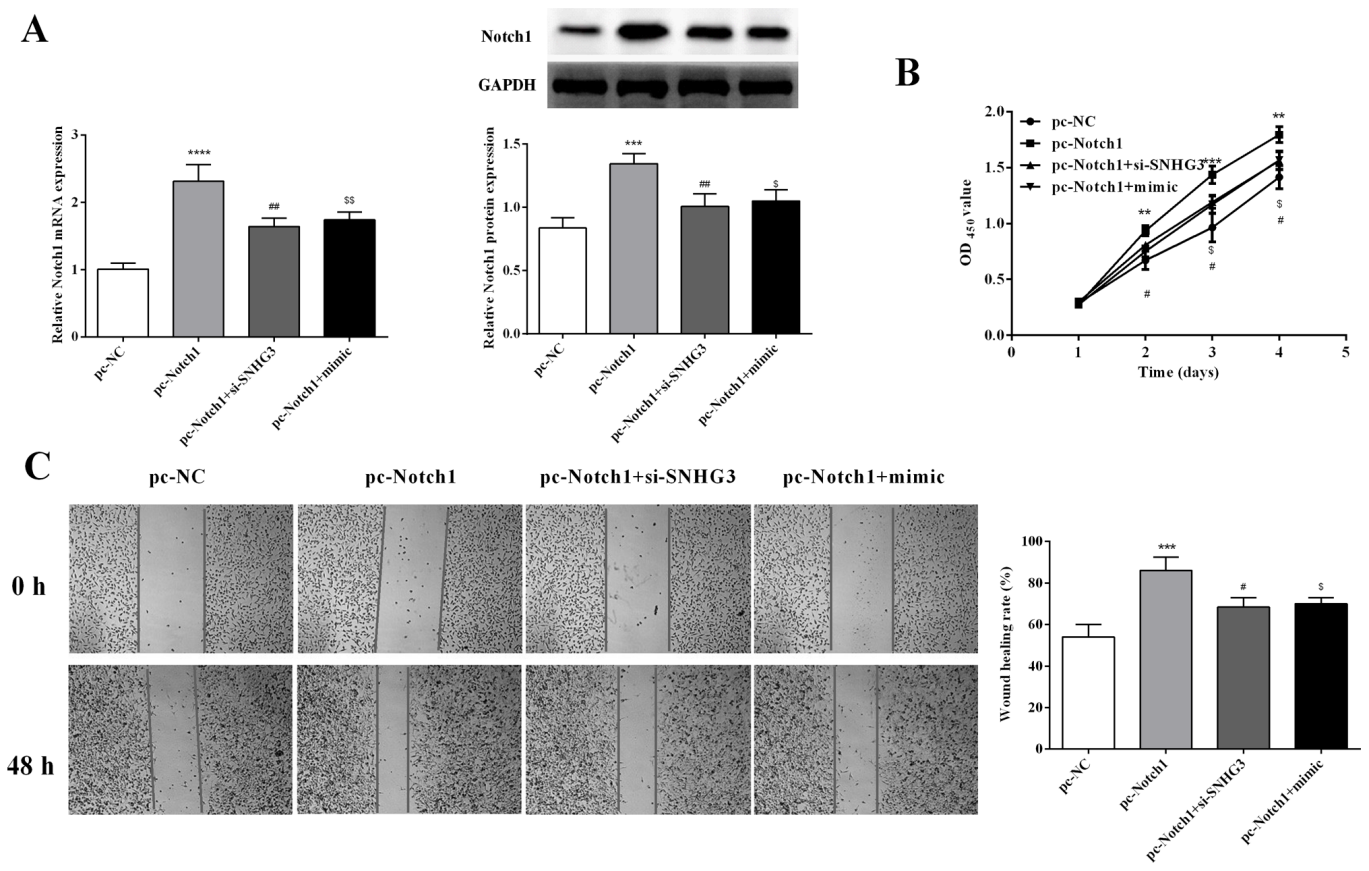
Notch1 overexpression overturns the suppressive effect of SNHG3 knockdown or miR-139-5p overexpression on cell proliferation and migration in OVCAR3 cells. (A) Notch1 expression in OVCAR3 cells. (B and C) Cell proliferation and migration were detected by CCK-8 and wound healing assays. **P<0.01, ***P<0.001 and ****P<0.0001 compared with pc-NC; ^#^P<0.05, ^##^P<0.01compared with pc-Notch1; $P<0.05, $$P<0.001 compared with the pc-Notch1 + si-SNHG3 group. Notch 1, Notch homolog 1, translocation-associated (Drosophila); SNHG3, small nucleolar RNA host gene 3; miR, microRNA; si-SNHG3, small interfering RNA targeting SNHG3; CCK-8, Cell Counting Kit-8; NC, negative control.

